# Supplementary figures and images for: A nonstructural protein encoded by a rice reovirus induces an incomplete autophagy to promote viral spread in insect vectors
Source: PLoS Pathog. 2022 May 9;18(5):e1010506. doi: 10.1371/journal.ppat.1010506 (PMC9119444; doi:10.1371/journal.ppat.1010506)

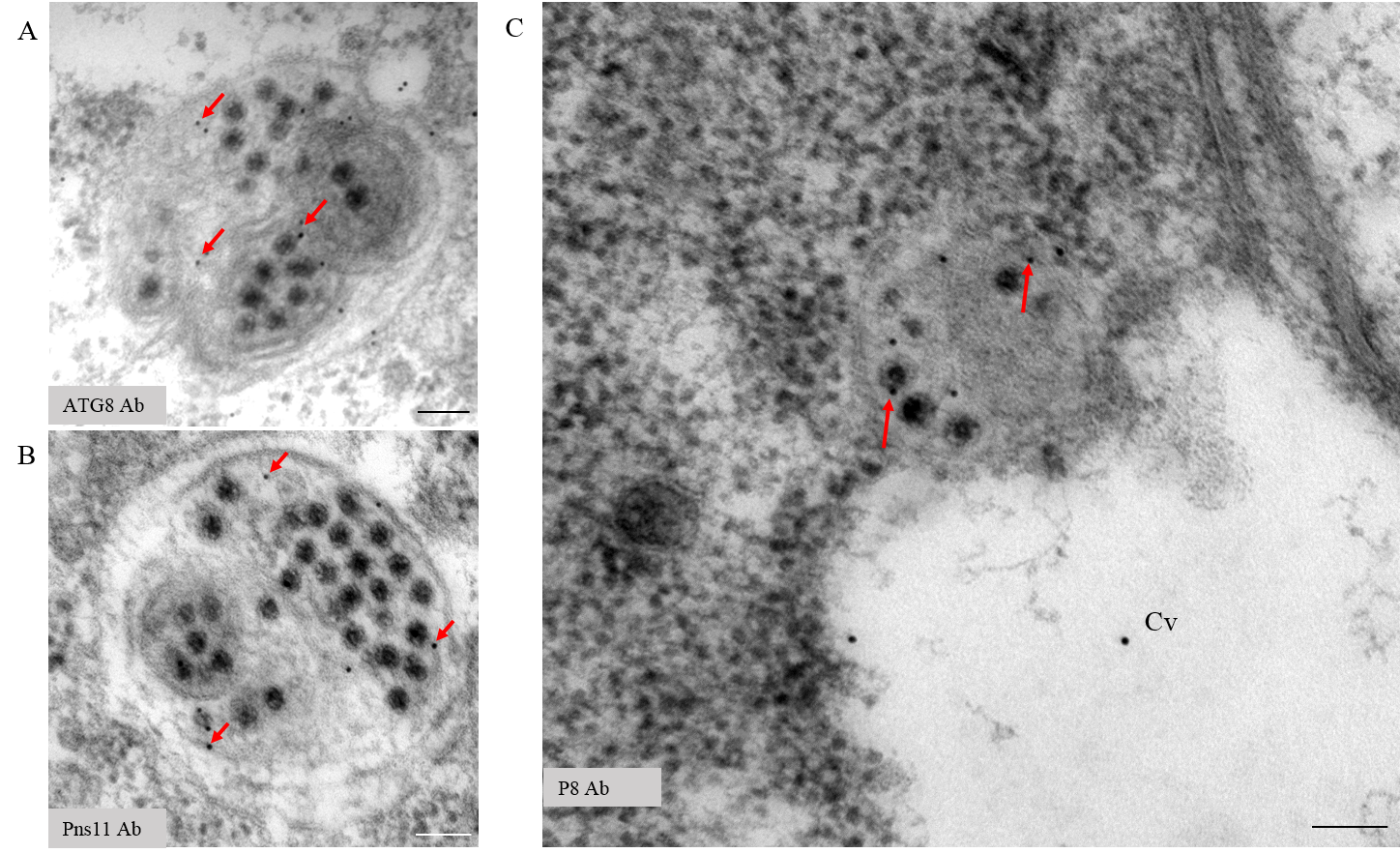

Supplement: S1 Fig — (A-C) Immunogold labeling of ATG8 (A), Pns11 (B) or P8 (C) in virus-containing autophagosomes in the midgut (A, B) or salivary gland (C). Virus-infected internal organs were immunolabeled with ATG8- (A), Pns11- (B) or P8- (C) specific IgG as the primary antibody, followed by treatment with 10-nm gold particle-conjugated IgG as the secondary antibody. Cv, cavity. Red arrows indicate gold particles. Bars, 100 nm. (TIF) [file ppat.1010506.s001.tif]

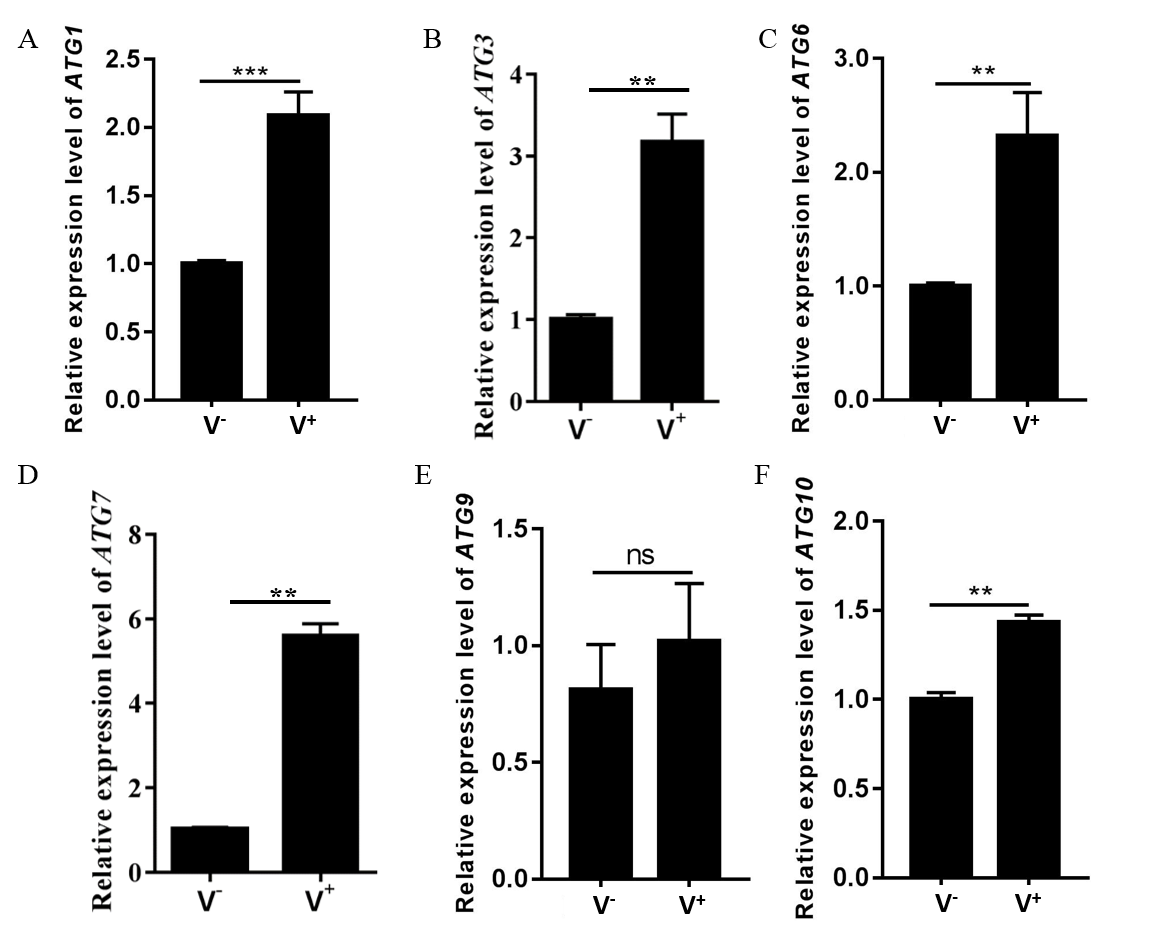

Supplement: S2 Fig — The relative transcript levels of ATG1 (A), ATG3 (B), ATG6 (C), ATG7 (D), ATG9 (E) and ATG10 (F) were detected by RT-qPCR assay. Data are presented as means ±SD from three independent experiments. Significance (**) was determined at P < 0.01. ns, not significant. (TIF) [file ppat.1010506.s002.tif]

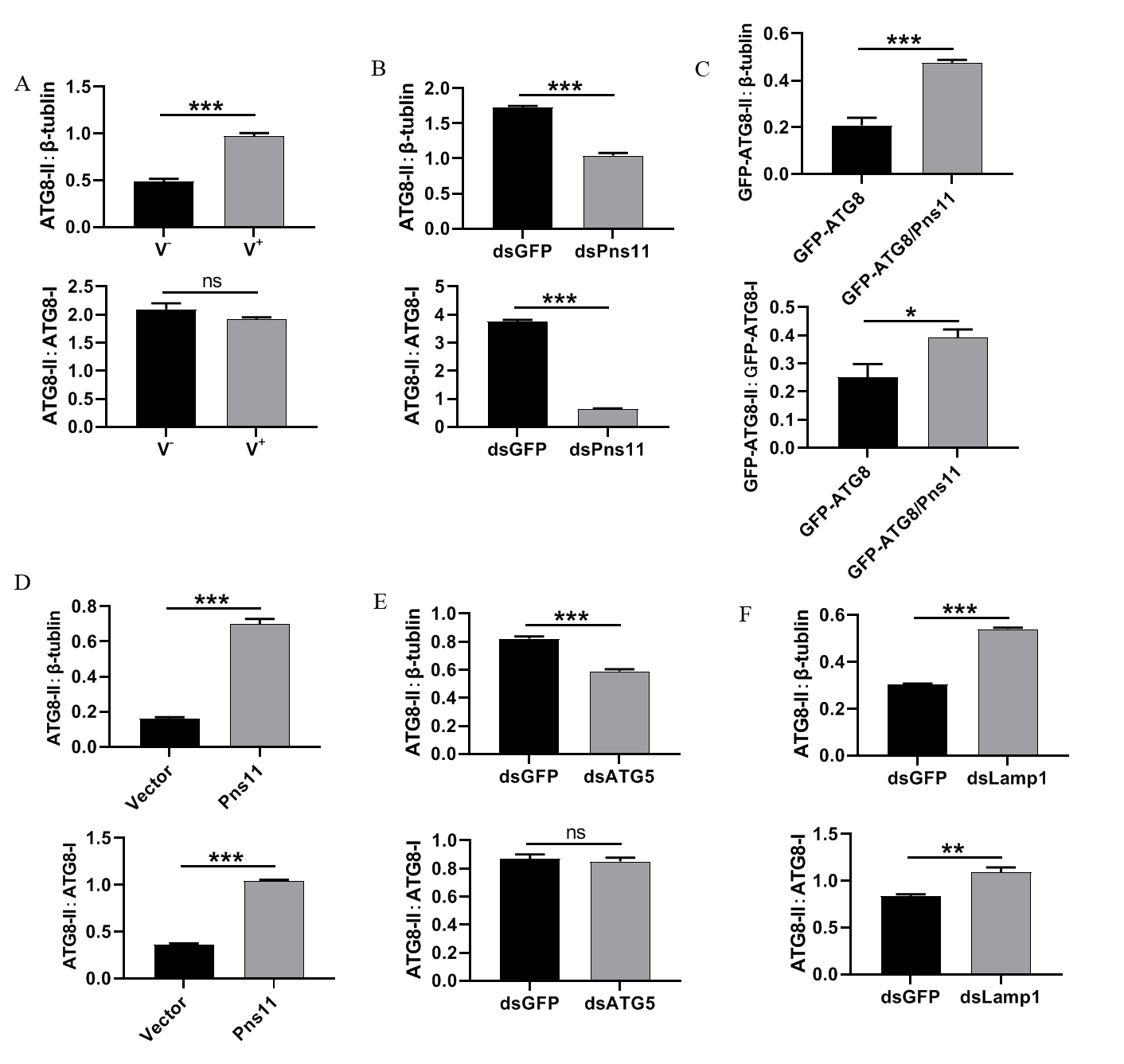

Supplement: S3 Fig — Relative accumulation levels were determined using ImageJ, and the ratios of ATG8-II to ATG8-I or Tubulin in Figs 1J (A), 3A (B), 3B (C), 5D (D), 5J (E) and 6L (F) were analyzed. Significance (*) was determined at P < 0.05. Significance (**) was determined at P < 0.01. Significance (***) was determined at P < 0.001. ns, not significant. (TIF) [file ppat.1010506.s003.tif]

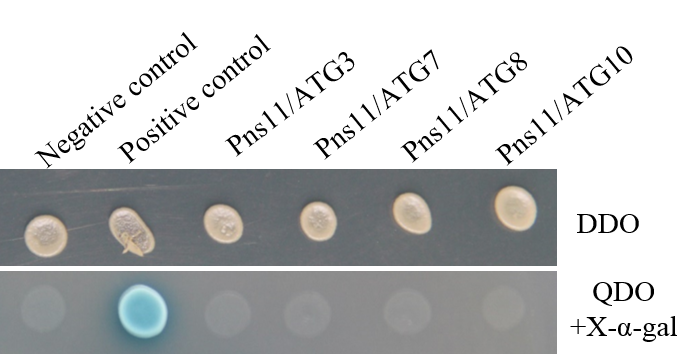

Supplement: S4 Fig — Interactions between Pns11 and ATG3, ATG7, ATG8 or ATG10 were detected by yeast two-hybrid assay. Transformants were plated on either DDO or QDO+X-α-Gal culture medium, and the pairs are labeled as follows: Pns11/ATG3, pGBKT7-Pns11/pGADT7-ATG3; Pns11/ATG7, pGBKT7-Pns11/pGADT7-ATG7; Pns11/ATG8, pGBKT7-Pns11/pGADT7-ATG8; Pns11/ATG10, pGBKT7-Pns11/pGADT7-ATG10; Positive control, pGBKT7-53/pGADT7-T; Negative control, pGBKT7-Lam/pGADT7-T. (TIF) [file ppat.1010506.s004.tif]
