# Supplementary material for: A nonstructural protein encoded by a rice reovirus induces an incomplete autophagy to promote viral spread in insect vectors
Source: PLoS Pathog. 2022 May 9;18(5):e1010506. doi: 10.1371/journal.ppat.1010506 (PMC9119444; doi:10.1371/journal.ppat.1010506)
Supplement: S1 Table — (DOC) [file ppat.1010506.s005.doc]

S1 Table Primers used in this study.

| Purpose | Construct | Primer sequences | | |
| --- | --- | --- | --- | --- |
|  |  | Forward |  | Reverse |
| Yeast two hybrid assay | pGBKT7- Pns11 | 5'-ATGGCCATGGAGGCCGAATTCATGGATGCGGACACTGAGCG-3' |  | 5'-CCGCTGCAGGTCGACGGATCCCTAGCTTTCAAGCTTCAATGAT-3' |
|  | pGADT7-ATG5 | 5'-GCCATGGAGGCCAGTGAATTCATGGCCTGTGATAGAGAGGTTTTG-3' |  | 5'-CAGCTCGAGCTCGATGGATCCTTATGAGTAGCCGACGCAGAGA-3' |
|  | pGADT7-ATG12 | 5'-GCCATGGAGGCCAGTGAATTCATGGCCGAGTGTGAATCACC-3' |  | 5'-CAGCTCGAGCTCGATGGATCCTTAGCCCCAAGCTTGACCG-3' |
|  | pGADT7-Lamp1 | 5'-GCCATGGAGGCCAGTGAATTCATGCGGGGTTCTCATCATCATC-3' |  | 5'-CAGCTCGAGCTCGATGGATCCTTACATGCTCAGATATCCCCGAGAC-3' |
| Baculovirus expression assay | pFastBac1-Pns11 | 5'-CCCACCATCGGGCGCGGATCCATGGATGCGGACACTGAGCG-3' |  | 5'-CTAGTACTTCTCGACAAGCTTCTAGCTTTCAAGCTTCAATGAT-3' |
|  | pFastBac1-P8 | 5'-CCCACCATCGGGCGCGGATCCATGAAATTCCAATACAAAGAAGA-3' |  | 5'-CTAGTACTTCTCGACAAGCTTAGCATCCCCGTAGACATTTTCA-3' |
|  | pFastBac1-ATG5-His | 5'-CCCACCATCGGGCGCGGATCCATGGCCTGTGATAGAGAGGTTTTG-3' |  | 5'-CTAGTACTTCTCGACAAGCTTTTAATGGTGATGGTGATGATGTGAGTAGCCGACGCAGAGA-3' |
|  | pFastBac1-ATG12-Flag | 5'-CCCACCATCGGGCGCGGATCCATGGCCGAGTGTGAATCACC-3' |  | 5'-CTAGTACTTCTCGACAAGCTTTTACTTATCGTCGTCATCCTTGTAATCGCCCCAAGCTTGACCG-3' |
|  | pFastBac1-GFP-  ATG8-1 | 5'-CCCACCATCGGGCGCGGATCCATGAGTAAAGGAGAAGAACTT-3' |  | 5'- CTTCTTTGTATTGGAATTTCATTTTGTATAGTTCATCCATGCCA -3' |
|  | pFastBac1-GFP-  ATG8-2 | 5'-TGGCATGGATGAACTATACAAAATGAAATTCCAATACAAAGAAG-3' |  | 5'-CTAGTACTTCTCGACAAGCTTTTAAGCATCCCCGTAGACATTTTC-3' |
|  |  |  |  |  |
| dsRNA assay | T7-GFP | 5'-*ATTCTCTAGAAGCTTAATACGACTCACTATAGGG*ACGTAAACGGCCACAAGTTC-3' |  | 5'-*ATTCTCTAGAAGCTTAATACGACTCACTATAGGG*AAGTCGTGCTGCTTCATGTG-3' |
|  | T7-ATG5 | 5'-*ATTCTCTAGAAGCTTAATACGACTCACTATAGGG*ATGGCCTGTGATAGAGAGGTTTTG- 3' |  | 5'-*ATTCTCTAGAAGCTTAATACGACTCACTATAGGG*TTATGAGTAG CCGACGCAGAGATGC -3' |
|  | T7-Pns11 | 5'-*ATTCTCTAGAAGCTTAATACGACTCACTATAGGG*ATGGATGCGGACACTGAGCGTG-3' |  | 5'-*ATTCTCTAGAAGCTTAATACGACTCACTATAGGG*TTCCTTATATGTAGAACTCCAT- 3' |
|  | T7-Lamp1 | 5'-*ATTCTCTAGAAGCTTAATACGACTCACTATAGGG*TATGGCTAGCGAATTGGCCC-3' |  | 5'-*ATTCTCTAGAAGCTTAATACGACTCACTATAGGG*AGTTGCCATTGCATGTTCCG- 3' |
| RT-qPCR assay | q-ATG5 | 5'-ACTCCAACCTCCAGAACCAT-3' |  | 5'-CCATTTCAAAGGCAGTCCAT- 3' |
|  | q-ATG8 | 5'-AGAAGTATCTGGTGCCCTCCG-3' |  | 5'-CTTCGTGGTGCTCCTGGTAGA- 3' |
|  | q-ATG12 | 5'-GAAGAAGAAGAAGTGGTCAGTAG-3' |  | 5'- AATCAGTTTTCCGTCAGTTTCGT-3' |
|  | q-p62 | 5'-GCGCTGCCTCGTCTGATTCCTCT-3' |  | 5'-GCTGGCGGACGGGATTTCA-3' |
|  | q-Lamp1 | 5'-AATCGAATGCGACTGTAAAAGGCC-3' |  | 5'-GGAGGTTCGGGCTTAGGCGT-3' |
|  | q-Pns11 | 5'- GGTGGTATTGATTGACGCATGT-3' |  | 5'- TCGGTCTCATCGTTCCCATCTT-3' |
|  | q- EF1 | 5'-CAGTGAGAGCCGTTTTGAG-3' |  | 5'- AGGGCATCTTGTCAGAGGGC-3' |
| Pull-down assay | pGEX-4T-ATG5 | 5'-GGATTTCACATATGTCCATGGCCTGTGATAGAGAGGTT-3' |  | 5'-CTCGAGTCGACCCGGGAATTCTTATGAGTAGCCGACGCAGAG- 3' |
|  | pGEX-4T-Pns11 | 5'-GGATTTCACATATGTCCATGGATGCGGACACTGAGCGT-3' |  | 5'-CTCGAGTCGACCCGGGAATTCCTAGCTTTCAAGCTTCAATGAT- 3' |
|  | pDEST17-Pns11 | 5'-GGGGACAAGTTTGTACAAAAAAGCAGGCTTCATGGATGCGGACACTGAGCGTG-3' |  | 5'-GGGGACCACTTTGTACAAGAAAGCTGGGTCGCTTTCAAGCTTCAATGATTCT-3' |
|  | pDEST17-ATG12 | 5'-GGGGACAAGTTTGTACAAAAAAGCAGGCTTCATGGCCGAGTGTGAATCACC-3' |  | 5'-GGGGACCACTTTGTACAAGAAAGCTGGGTCGCCCCAAGCTTGACCGAGGC-3' |
|  | pDEST17-Lamp1 | 5'-GGGGACAAGTTTGTACAAAAAAGCAGGCTTCATGCGGGGTTCTCATCATCATC-3' |  | 5'-GGGGACCACTTTGTACAAGAAAGCTGGGTCCATGCTCAGATATCCCCGAGA-3' |

The recombination site sequence were underlined and the T7 promoter sequence were marked in italics.
