# Supplementary material for: A nonstructural protein encoded by a rice reovirus induces an incomplete autophagy to promote viral spread in insect vectors
Source: PLoS Pathog. 2022 May 9;18(5):e1010506. doi: 10.1371/journal.ppat.1010506 (PMC9119444; doi:10.1371/journal.ppat.1010506)
Supplement: Attachment — Submitted filename: Response to Reviewers.doc [file ppat.1010506.s006.doc]

**Point by point to Comments**

We would like to thank the editor and three expert reviewers for their insightful comments and suggestions on the manuscript, as these comments and suggestions led us to an improvement of the manuscript. Our revision reflects all suggestions and comments. Detailed responses to the editors and reviewers are given below.

Reviewer's Responses to Questions

All reviewers note that new information is presented in this manuscript, but all reviewers raise important concerns. I agree with many of their concerns.

Reviewer 1 raises an important point about interpretation of autophagosome delivery of virus particles. This is based on your TEM data and needs to be clarified.

Response: We have supplemented the experiment about dsATG5 treatment inhibited viral transmission in Fig. 5K, which suggested that inhibited the formation of autophagosome effected the spread of virus from midgut to salivary glands.

Reviewer 2 recommends major revision, mostly regarding the specific language used in the manuscript and the detail/clarity of the figure labeling. Some examples are given.

Response: We have carefully checked and revised the figure labeling.

Reviewer 3 recommends rejection, and raises a number of important points which must be addressed. The reviewer raises the points in Figures 1G and 1H about Lamp1 reductions. The RT-qPCR data in 1G do not support this, and 1H shows no statistics to document the claim. See your comments on lines 156 and 157. This reviewer also raises the point concerning statements about Lamp1 on the lysosomal membrane on Line 257.

Response: In Fig. 1G, we detected the mRNA level of Lamp1. In Fig. 1H, we detected the protein accumulation levels of most densely N-glycosylated Lamp1 with *Mr* ~120 kDa. We further found that Pns11 were not localized with lysosome in RGDV infected cells. Thus, we supposed that Pns11 blocked the N-glycosylation, but not the expression of Lamp1 by interaction and decreased the N-glycosylated Lamp1 in lysosome. We have revised the results section about Pns11 interacted with Lamp1.

You also make global statements about the significance of your findings here with other insect vectored plant viruses, for example see the last line of the abstract and even the last line of the discussion suggests that this is a conserved property. This is over-reaching what you present here.

Response: We have revised the description.

Reviewer's Responses to Questions

Part I - Summary

Please use this section to discuss strengths/weaknesses of study, novelty/significance, general execution and scholarship.

Reviewer #1: The manuscript is about inducing an incomplete autophagy by the rice gall dwarf virus to increase virus spread in its insect vector. Autophagy is considered as an important defense mechanism against pathogens evading, and how viruses have evolved to subvert this mechanism for their benefit is an interesting topic in the field of virology. This fact that viruses target the autophagy as a defense mechanism of host cells is not new and it has been shown for some animal and plant viruses. However, most of these results were illustrated in vitro. This manuscript shows this phenomenon in vivo which is interesting. The authors showed that one of the viral non-structural proteins (Pns11) is involved in formation of autophagosomes by interacting with ATG5. Additionally, Pns11 downregulates the expression of Lamp1 to prevent degradation of autophagosomes by lysosome. The manuscript written well, and results presented nicely.

Response: Thank you for your positive envaluation of our work.

My main question/concern is about the conclusion/claim that the authors has made about the role of autophagosomes in virus spread by the data presented in this manuscript. Although the EM shows that viral particles associated with autophagosomes are released from midgut to hemocoel and the presence of the autophagosomes that contain viral particles was also showed in salivary glands, I am concerning/wondering if this is enough to conclude and claim that autophagosomes facilitate the virus transportation across insect membrane barriers. This is a strong statement. Do authors have any plans to use or suggest any other methods to validate this conclusion/claim?

Response: Thanks for your valuable suggestions. In order to successfully transmit virus to rice plants, the persistent plant viruses must move through the insect vector, from the midgut epithelium to other organs including the salivary glands, and overcome various tissue or membrane barriers with in the insect. We have supplemented the results that dsATG5 treatment inhibited viral transmission in Fig. 5K. When inhibited the formation of autophagosomes by dsATG5, viral transmission rate was significantly decreased, which concluded that autophagosomes facilitate viral transpiration across insect midgut or salivary glands release barriers.

Reviewer #2: This study described the discovery and characterization of enzymatically inactive autophagosomes induced by a virus (rice gall dwarf reovirus) in the cells of the insect that is both a host of this virus, and a transmitting vector. These “incomplete” autophagosomes were incapable of degrading the virus particles they enclosed, but were instead repurposed by the virus for its own spread inside the insect. The authors established that such autophagosomes were induced by nsP11, one of the RGDV-encoded proteins, by its direct interaction with ATG8. Furthermore, inhibiting their formation by down-regulating nsP11 or ATG8 expression compromised RGDV infection of the insect.

Overall the manuscript is reasonably well written, and the data support the conclusions drawn. The quality of the figures is generally good but better labeling could improve the readability (see below).

Response: Thank you for your positive envaluation of our work.

The language needs to be more carefully proof-read. Numerous examples are provided below, though they are by no means comprehensive.

Response: We have carefully checked and revised the language in the full text.

Reviewer #3: The manuscript by Jia et al provides evidence to demonstrate the role of a nonstructural protein, Pns11, of the rice gall dwarf virus (RGDV) in controlling autophagosome formation in leafhoppers through its association with an autophagy-related protein ATG5, and the apparent correlation between this interaction and the conversion of the ATG8-I to ATG8-II complex (a feature key to the formation of autophagosomes). The aforesaid interaction also is shown to be associated with another autophagy-related protein ATG12, an autophagic adapter protein p62, and a lysosomal protein Lamp1. The manuscript also presents results showing the direct interaction between RGDV Pns11 and Lamp1 and the lack of interaction between RGDV P8 (the outer capsid protein) and Lamp1 as evidence that the RGDV Pns11-Lamp1 interaction prevents the formation of autolysosomes and degradation of RGDV.

The manuscript shows interesting associations among RGDV Pns11, ATG5, p62 and Lamp1 at the molecular level, and implicates the role of RGDV Pns11 in eliciting changes in the ATG8 complexes. The TEM micrographs showing the presence of RGDV in double-membraned autophagosomes within the midgut and salivary glands are conspicuous and of high quality. However, there are several technical gaps in the studies that give some pause to the conclusions made, indicating that the conclusions may be too preliminary. The manuscript also lacks important background information and is scattered with many reporting and editorial errors on top of missing information in specific sections of the results.

Response: Thank you for your positive envaluation of our work. We have added important background information, supplemented dsLamp1 treatment experiment in Fig 6 and revised the reporting or editorial errors in the results.

Part II – Major Issues: Key Experiments Required for Acceptance

Please use this section to detail the key new experiments or modifications of existing experiments that should be absolutely required to validate study conclusions.

Generally, there should be no more than 3 such required experiments or major modifications for a "Major Revision" recommendation. If more than 3 experiments are necessary to validate the study conclusions, then you are encouraged to recommend "Reject".

Reviewer #1: (No Response)

Reviewer #2: None.

Reviewer #3: 1) Introduction: There needs to be some more background information about phagosome formation with details describing what is known (or unknown) about phagosome formation in animal and insect systems. Questions such as ‘how is ATG8-II related to ATG8-I?’ and ‘what is the basis for the difference in Mr between ATG8-II and ATG8-I as seen in many of the figures (e.g. Figs. 1H, 2B, 3A and 3B, 5B, 5J)?’ need to be addressed with information. Information about the viral encoded proteins is also needed since tests were done to determine if several non-structural proteins of the virus interacted with autophagosomes (L171). What functions are these ns proteins involved in? Another example of the lack of background information is that of the structural protein RGDV P8. Anti-P8 IgGs (Materials and Methods L402) were used to localize RGDV in the intestines (e.g. in Fig. 1K); why is the presence of P8 representative of the virus? One has to reach as far in as the third result section (corresponding to the results in Fig. 3) to find out that P8 is the RGDV outer capsid protein.

Response：We have added the phagosome formation in animal and insect in introduction. We have supplemented supplementary figure 3 to display the ratios of ATG8-II to ATG8-I or Tubulin controls of Figs. 1J, 3A, 3B, 5D, 5J and 6L, and added the molecular weight of ATG8-I and ATG8-II in Figs. 1J, 3A, 3B, 5D, 5J and 6L. We have added the background information of the structural protein RGDV P8 and nonstructural proteins (Pns4, Pns7, Pns9, Pns10, Pns11 and Pns12) in introduction.

2) Results associated with Fig. 1: it is mentioned in the introduction that the coupling of ATG12 and ATG5 are involved in heterodimer formation on the phagophore. Therefore, it makes sense that the level of ATG12 also ought to be determined in viruliferous and non-viruliferous insects. However, this was not done, and no explanation was provided.

Response: We have added the relative transcript level of ATG12 in Fig. 1G, deleted repetitive pictures of Fig 1C and 1E and adjusted the layout of Fig 1.

3) Results in Fig. 1H are consistent with the writing in L149-L151, but there is no indication here or in L377 (M&M) if quantified, equal amounts of total RNA was used for the samples. Neither was there an indication of how many insects were used for total RNA extraction (in M&M).

Response：We have added the quantity of insects that used for total RNA extraction in Materials and Methods section. In Fig. 1, total RNAs or total proteins from 50 viruliferous or nonviruliferous intact insects were extracted and used for RT-qPCR and Western blot assay.

4) The experimental results describing the increased accumulation of GFP-ATG8-II in SF9 cells co-transfected with GFP-ATG8 and Pns11 or with GFP-ATG8 alone (L198-L199, Fig. 3A) are not convincing. To begin with, there appears to be more GFP-ATG8-I in SF9 cells co-transfected with GFP-ATG8 and Pns11 than in SF9 cells transfected with GFP-ATG8. It would be more appropriate to determine the ratio of GFP-ATG8-I to GFP-ATG8-II in SF9 cells expressing Pns11 and GFP-ATG8 vs that of SF9 cells expressing GFP-ATG8 alone.

Response：Thanks for your valuable suggestions and we have added the ratio of GFP-ATG8-I to GFP-ATG8-II in Fig. S3B and 3C.

5) The Y2H and pull-down assays in Fig. 5 clearly show the association between Pns11 and Lamp1. However, one cannot rule out that within the midgut epithelia, the interaction could well be occurring at multiple locations in the cell cytoplasm and not just in the lysosomal membrane. Therefore, this ought to be tested in an experiment to determine if Pns11 and Lamp1 are co-localized in the lysosomes within the midgut epithelia as well as in the salivary glands of viruliferous insects. It is also unclear what Fig. 6E is meant to convey. It seems the conclusion drawn from the results is that the interaction of Pns11 and Lamp1 in SF9 cells leads to the degradation of Lamp1; hence, by inference, the same phenomenon is also occurring in the midgut of viruliferous leafhoppers. This conclusion is too presumptive. To be able to make a solid conclusion that the association of Pns11 and Lamp1 prevents the formation of autolysosomes, one would also need to perform an experiment to knockdown Lamp1 using dsLamp1. With a transient loss of Lamp1, one might expect to see an increase in RGDV and the number of autophagosomes in the midgut of viruliferous insects. In the absence of evidence supported by the additional experiments mentioned above, the model in Fig. 7 should be tempered by a measure of caution based on the preliminary nature of the key conclusion being presented to the reader.

Response：Thanks for your valuable suggestions. We have supplemented the location of Pns11and lysosome in leafhopper midgut epithelia in Fig. 6F-6H. The results showed that Pns11 did not co-localized with lysosome and RGDV infection significantly decreased the number of lysosomes in cells. Lamp1 is a most densely N-glycosylated lysosomal membrane glycoproteins. We speculated the interaction between Pns11 and Lamp1 in cells might inhibit the N-glycosylation of Lamp1 and further effected the decoration of Lamp1 in lysosomes and the stabilization of lysosome.

We have added the experiment to knockdown Lamp1 using dsLamp1. The results were displayed in Fig. 6I-O. After treated with dsLamp1 for 6 days, the expression of Lamp1 decreased, while RGDV Pns11 and ATG5 were significantly increased. The protein accumulation levels of RGDV Pns11, ATG5, ATG8, p62 were significantly increased, while that of Lamp1 was decreased. These results suggested that inhibited the formation of lysosome also facilitate the virus transportation by virus-containing autophagosome.

We have adjusted the model in Fig 7.

6) Discussion L346-L348 presents an over-interpretation of the results. The authors have only shown immuno-EM data (micrographs) suggesting the movement of phagosomes between gut epithelial junctions, and EM micrographs of membranous vesicles fused with the APL of the salivary gland. Therefore, a more appropriate conclusion ought to be “…..to form membranous structures implicated to be involved in viral spread within the regions that we have examined”.

Response: Thanks for your valuable suggestions and we have followed.

Part III – Minor Issues: Editorial and Data Presentation Modifications

Please use this section for editorial suggestions as well as relatively minor modifications of existing data that would enhance clarity.

Reviewer #1: - Page 3 line 77, it should be ATG8-I.

Response: We have revised it.

- Page 8, lines 237-239: Figure 5A shows the virus (Pns11) gene expression level. However, the sentence in the text does not mention this at all. It only talks about the ATG5 and ATG8. Either add the virus part or remove figure 5A in the parenthesis. The same for the following sentence. Figure 5A needs to be added in the parenthesis. As well

Response: Thanks for your valuable suggestions and we have followed.

- Page 8, lines 235 and 247: What was the reason to use nymphs in two different life stages (2nd and 3rd) for microinjection?

Response: In method section, we have described that second instar nymphs were microinjected with 0.5 μg/μl dsRNAs targeting their respective sequences (dsATG5, dsPns11 or dsGFP) after feeding on RGDV-infected rice plants for 1 day. In line 247, the nymphs is also second star nymphs. We have revised it.

- Page 11 lines 340-343: Add the “by an unknown mechanism” to the end of the sentence.

Response: We have revised it.

- Figure S2: ATG3 should be italic.

Response: We have revised it.

Reviewer #2: Fig. 1. What is the purpose of panel A? Is it a control section derived from a healthy insect, or lower magnification of B? It would be helpful to add a little more details here, and label the ruler size. In B to F, instead of using arrows of different colors, why not simply label them (one each) as “gold particle”, “double membrane”, “virion”? Also add the antibody used on respective panels (e.g. C – RGDV P8 Ab).

Response: Thanks for your valuable suggestions and we have followed. Fig. 1A is a control section derived from a healthy insect. We have labeled the arrows with “gold particle”, “double membrane” or “virion”.

Ln37, delete “in vivo” – redundant.

Ln38, add coma after “dwarf virus”

Ln39, change “within” to “of the”

Ln41-42, “viral nonstructural protein Pns11 induces autophagy and embeds itself in the autophagosome membrane.”

Ln43, delete “the development of”.

Ln47-48, “……to induce the formation of autophagosomes, the latter then enclose virions, and facilitate viral spread inside the insect bodies”.

Response: Thanks for your valuable suggestions and we have followed above.

Ln48-50, be specific – how does Psn11 causes Lamp1 down-regulation?

Response: In our results, we detected the RGDV infection mainly caused the protein accumulation of the most densely N-glycosylated Lamp1 decrease, but not effected the mRNA levels of Lamp1. Predicted molecular weight of RdLamp1 protein is about 38.2 kDa, and that of the most densely N-glycosylated Lamp1 is about 120 kDa. We speculated the interaction between Pns11 and Lamp1 in cells might inhibit the N-glycosylation of Lamp1 and further effected the decoration of Lamp1 in lysosomes and the stabilization of lysosome.

Ln54, delete “can”

Ln57, either “and” or “thus” has to go. Redundant.

Ln60, “mediating the down-regulation expression” – “down-regulating the expression” (same in Ln49).

Ln139, delete “characteristics”; add coma at the end of the line.

Response: Thanks for your valuable suggestions and we have followed above.

Ln140, “virus-infected” – “RGDV-infected”

Ln142, end, delete “were”

Ln143, add “an” before “RGDV-specific”.

Ln149, change “ATGs expression” to “the expression of ATGs” (to be more accurate, it should be “the mRNA levels of several ATGs”

Ln150, changed “increased” to “induced”; between “while” and “p62”, and “that of”

Ln151, delete “expression”; add comma after “stable”.

Ln152, “band” should be “protein level”.coma

Response: Thanks for your valuable suggestions and we have followed above.

Ln154, why “endogenous”? does the virus also encode an ATG8 homolog?

Response: We have deleted “endogenous”.

Ln166, “the component” should be “a component”

Response: Thanks for your valuable suggestion and we have followed.

Reviewer #3: L170-L171: ‘immunoelectron microscopy was first employed to investigate which viral nonstructural proteins (Pns4,….or Pns 12) of RGDV localized to autophagosomes’. What were the results for Pns-4, -7, -9, -10, and -12)? Even if the results were negative, they should be mentioned.

Response: We have added the description about Pns-4, -7, -9, -10, and -12 in result section.

L189-L191: “Taken together, RGDV can…….finally facilitating viral transmission by insect vectors (Fig 2F and 2G)’. These two figures are illustrative drawings of the presumed circulative pathway taken by the virus. To conclude that the autophagosomes facilitate viral transmission, one would have to link the RNAi knockdown studies of ATG5 to RGDV transmission study outcomes, but these studies are not reported in this manuscript.

Response: Thanks for your valuable suggestion and we have added the results about RGDV transmission in Fig. 5K.

L236: “with a mixture of purified viruses”. How were the viruses purified? This is not mentioned in the Materials and Methods.

Response: We have revised the description in L236. In this study, we did not microinject the second instar nymphs of *R. dorsalis* with purified viruses. We only microinjected the second instar nymphs of *R. dorsalis* with dsRNAs after feeding on RGDV-infected rice plants for 1 day. This has mentioned in the Materials and Methods.

MATERIALS AND METHODS

Sequence information of all primers used in the study should be provided, examples include those used for making the bacmid constructs (L429), the constructs used for GST pull-down assays (L452), and the constructs used for Y2H analysis (L416).

Response: We have added the sequence information of primers used in this study in Supplementary table 1.

L387: How were total proteins extracted from the insects?

Response: We have added the method of total proteins extracted in Western blot assay. Briefly, 50 insects were ground to a powder in liquid nitrogen and then dissolved with 0.2 mL lysis buffer (0.5 M phosphate, 0.5 M NaCl, 0.1% Tween-20, 0.1% NP-40, 1 mM Phenylmethanesulfonyl fluoride, 0.1% β-mercaptoethanol, 10 mM Tris-HCl, pH 8.0) and incubated 10 min at 4°C. After centrifugation (12,000 rpm, 20 min at 4°C), the supernatant was transferred to a new tube, and the protein concentration was measured using the Bradford method [22]. The crude protein was stored at −80°C until use.

L432: Information on the construction of the recombinant bacmid constructs is needed.

Response: We have added the information on the construction of the recombinant bacmid constructs in methods section.

L440: Information on the construction of GFP-ATG8 is needed.

Response: We have added the information on the construction of GFP-ATG8 in methods section.

L447-L450: There is a lot of overlapping materials and methods here and in L395 (“Examination of autophagy by immunofluorescence microscopy”) that can be consolidated.

Response: we have checked carefully. These two methods are different and could not be consolidated.

L480: for all experiments that involved the use of statistical analyses, the number of replicates used in the studies need to be provided either in the figure legend or in the text.

Response: We have carefully checked and added the number of replicates in the figure legend.

FIGURE LEGENDS

L617-L618: Figure 1 legend. What are the green arrows pointing at?

Response: The green arrows pointing at viron. We have label it with “viron”

L630: Figure 1 legend. How many times was the experiment involving the observation of 10 cells repeated?

Response: The experiment involving the observation of 10 cells repeated three times.

L638-L640: this is a repetition of the previous.

Response: We have deleted this sentence.

L644: Figure 2E. The panels in Fig. 2E are micrographs of the insect salivary glands without any immunogold labeling unlike in Fig. 2C (micrographs of the midgut invaded by RGDV). Without the immunolabeling, it is difficult to be convinced that those autophagosomes contained RGDV.

Response: We have supplemented a micrograph of the insect salivary glands that labeling with immunogold on autophagosomes contained RGDV in Fig. S1D.

L650-L653: what does “VM” represent in Fig. 2C?

Response: VM represent visceral muscle. We have added it in figure 2 legend.

L679-L680: antibodies to these proteins were used i.e. anti-His-rhodamine, anti-Flag-rhodamine or anti-Pns11-rhodamine.

Response: Thanks for your valuable suggestions and we have followed.

L697: Figure 5. What is the mock treatment? Based on the results, it looks like the mock treatment refers to non-viruliferous insects that were not subjected to any dsRNA treatment.

Response: The mock treatment refers to nonviruliferous insects that were not subjected to any dsRNA treatment.

L710-L713: Figure 6. What do the white arrows indicate?

Response: White arrows indicate Lamp1-positive lysosomes. We have added it in figure 2 legend.

A SELECTION OF EDITORIAL ISSUES/MISTAKES

L274: “positive RNA viruses”

Response: We have revised it as positive-strand RNA viruses.

L283: “infection in the insect intestinal, disseminate…”

Response: We have revised it as “infection in the insect intestinal epithelium, disseminate…”.

L345: “we demonstrates”

Response: We have revised the description in method section.

L370: “antibodies were then directly conjugated FITC”

Response: Thanks for your valuable suggestions and we have followed.

L388 and L392: “The bands were then transferred...” What bands? Please use the proper technical term to describe proteins separated by SDS-PAGE.

Response: We have revised the description in method section as “….were extracted. Protein samples were separated by SDS-PAGE and transferred onto polyvinylidene difluoride (PVDF) membrane”.

L463: “In vivo knocking down ATG5”

Response: We have deleted in vivo.

L464-L465: “..the functional roles of ATG5 or Pns11 in the autophagy inducing, the in vivo knocking down assay was conducted”

Response: We have revised this sentence as “To determine the functional roles of ATG5 or Pns11 during the induction of autophagy, the knocking down assay was conducted.”

Figure 7: “Autolysosome”

Response: We have revised it.
