# Supplementary material for: A nonstructural protein encoded by a rice reovirus induces an incomplete autophagy to promote viral spread in insect vectors
Source: PLoS Pathog. 2022 May 9;18(5):e1010506. doi: 10.1371/journal.ppat.1010506 (PMC9119444; doi:10.1371/journal.ppat.1010506)
Supplement: Attachment — Submitted filename: Response to Reviewers-2nd.doc [file ppat.1010506.s007.doc]

**Point by point to Comments**

Thanks for the comments on the manuscript, which would lead us to an improvement of the manuscript. Our revision reflects all suggestions and comments. Detailed responses to comments are given below.

The authors have done a good job in responding to many of the suggestions of reviewers. However, in one area, addressed by both reviewers 2 and 3, they did not do an adequate job. The writing in several sections is still not carefully done. The authors need to have an expert correct the language mistakes throughout this manuscript, including the figure legends. For example just in the abstract, author summary and first paragraph of the introduction, here are the numerous minor language errors:

Line 50, membranes should be plural

51, remodel should be plural, insert the, it should say “the insect vector”.

Line 61, membrane should be plural

Line 76, ATG 8-1 which is…insert which as shown

Line 77, conjugate should be conjugated

Line 78, membranes should be plural

Line 81, membranes should be plural

Line 83, anautolysosomes should be autolysosomes

Line 84, protein should be plural

Line 85, should be “and protect”

Line 86, delete the word “was”, and consist should be plural

Line 87, insert “is”….and is

Line 89 is an improper sentence.

Response: Thanks for your valuable suggestions and we have carefully checked and revised English in the full text.

There are many more of these simple errors throughout the manuscript which must be corrected.

Response: We have carefully checked and revised the language in the full text.

Other comments:

On line 209 - 215 the authors present data showing reduction of GFP-ATG8 when it is expressed by a baculovirus in Sf9 cells with pns11, they say increased accumulation of GFP-ATG8 and conversion to GFP-ATG8-II (Fig 3A). But for 3B they say increased expression, I think expression is the wrong word and accumulation/conversion are correct.

Response: Thanks for your valuable suggestions and we have followed.

Somewhat similarly to above, on lines 247 – 261, they do additional experiments with dsRNA RNA-I against ATG5. I think the data are good, but I think they use some incorrect words. I think they see decreased ATG8 and ATG8-II accumulation, I don’t think they see an effect directly on conversion of ATG8 to ATG8-II. If there is less ATG8, there of course will be less accumulation of ATG8-II. Thus I would delete the word conversion.

Response: Thanks for your valuable suggestions and we have followed.

For the data presented in lines 263 – 273, they argue that targeting pns11 by pns11 dsRNA-RNAi has the specific effects on levels of ATG5, ATG8-I/ATG8-II conversion, and the average number of ATG8-Pns11 puncta in midgut epithelial cells, and RGDV transmission. This is true, but it probably is not a result of specifically knocking down pns11, but by knocking down the pns11 RNA they are inhibiting RGDV replication and accumulation. They need to address this.

Response: Thanks for your valuable suggestions and we have revised the description.

Line 308, all viruses are intracellular

Line 388, change conclude to speculate.

Response: Thanks for your valuable suggestions and we have followed.
